# The Impact of AVATAR Therapy on Voice Hearing in Everyday Life: ESM Outcomes of the AVATAR2 Trial

**DOI:** 10.1093/schbul/sbaf100

**Published:** 2025-12-14

**Authors:** Clementine J Edwards, Robin Smith, Ginette Lafit, Thomas Ward, Richard Emsley, Mar Rus-Calafell, Inez Myin-Germeys, Emmanuelle Peters, Sandra Bucci, Thomas K Craig, Gillian Haddock, Hannah Ball, Miriam Fornells-Ambrojo, Amy Hardy, Hamish McLeod, Andrew I Gumley, Jeffrey McDonnell, Alice Montague, Moya Clancy, Mark Huckvale, Philippa Anne Garety

**Affiliations:** Department of Psychology, Institute of Psychiatry, Psychology & Neuroscience, King’s College London, London, United Kingdom; South London and Maudsley NHS Foundation Trust, London, United Kingdom; Department of Psychology, Institute of Psychiatry, Psychology & Neuroscience, King’s College London, London, United Kingdom; Research Group of Quantitative Psychology and Individual Differences, Faculty of Psychology and Educational Sciences, Methodology of Educational Sciences Research Group, KU Leuven, Leuven, Belgium; Department of Psychology, Institute of Psychiatry, Psychology & Neuroscience, King’s College London, London, United Kingdom; South London and Maudsley NHS Foundation Trust, London, United Kingdom; Department of Biostatistics and Health Informatics, Institute of Psychiatry, Psychology and Neuroscience, King’s College London, London, United Kingdom; Department of Psychology, Institute of Psychiatry, Psychology & Neuroscience, King’s College London, London, United Kingdom; Mental Health Research and Treatment Center, Faculty of Psychology, Ruhr-Universität Bochum, Bochum, Germany; Department of Neurosciences, Center for Contextual Psychiatry, KU Leuven, Leuven, Belgium; Department of Psychology, Institute of Psychiatry, Psychology & Neuroscience, King’s College London, London, United Kingdom; South London and Maudsley NHS Foundation Trust, London, United Kingdom; Division of Psychology and Mental Health, School of Health Sciences, University of Manchester and the Manchester Academic Health Sciences Centre, Manchester, United Kingdom; Greater Manchester Mental Health NHS Foundation Trust and the Manchester Academic Health Sciences Centre, Manchester, United Kingdom; South London and Maudsley NHS Foundation Trust, London, United Kingdom; Department of Health Service and Population Research, Institute of Psychiatry, Psychology and Neuroscience, King’s College London, London, United Kingdom; Division of Psychology and Mental Health, School of Health Sciences, University of Manchester and the Manchester Academic Health Sciences Centre, Manchester, United Kingdom; Greater Manchester Mental Health NHS Foundation Trust and the Manchester Academic Health Sciences Centre, Manchester, United Kingdom; Division of Psychology and Mental Health, School of Health Sciences, University of Manchester and the Manchester Academic Health Sciences Centre, Manchester, United Kingdom; Greater Manchester Mental Health NHS Foundation Trust and the Manchester Academic Health Sciences Centre, Manchester, United Kingdom; Research Department of Clinical, Educational and Health Psychology, University College London, London, United Kingdom; North East London NHS Foundation Trust, London, United Kingdom; Department of Psychology, Institute of Psychiatry, Psychology & Neuroscience, King’s College London, London, United Kingdom; South London and Maudsley NHS Foundation Trust, London, United Kingdom; Institute of Health and Wellbeing, School of Mental Health and Wellbeing, University of Glasgow, Glasgow, United Kingdom; NHS Greater Glasgow and Clyde, Glasgow, United Kingdom; Institute of Health and Wellbeing, School of Mental Health and Wellbeing, University of Glasgow, Glasgow, United Kingdom; NHS Greater Glasgow and Clyde, Glasgow, United Kingdom; Research Department of Clinical, Educational and Health Psychology, University College London, London, United Kingdom; North East London NHS Foundation Trust, London, United Kingdom; South London and Maudsley NHS Foundation Trust, London, United Kingdom; Research Department of Clinical, Educational and Health Psychology, University College London, London, United Kingdom; North East London NHS Foundation Trust, London, United Kingdom; Institute of Health and Wellbeing, School of Mental Health and Wellbeing, University of Glasgow, Glasgow, United Kingdom; NHS Greater Glasgow and Clyde, Glasgow, United Kingdom; Department of Speech, Hearing and Phonetic Sciences, University College London, London, United Kingdom; Department of Psychology, Institute of Psychiatry, Psychology & Neuroscience, King’s College London, London, United Kingdom

**Keywords:** AVATAR therapy, hearing voices, digital therapy, psychosis, randomized controlled trial, experience sampling methodology

## Abstract

AVATAR therapy involves facilitated dialogs between a voice hearer and a digital embodiment of their distressing voice (“the avatar”). We conducted a multi-site single-blind randomized controlled trial to evaluate the efficacy of brief (AV-BRF) and extended (AV-EXT) forms of AVATAR therapy, compared with treatment as usual (TAU) alone (AVATAR2). This study reports the data from experience sampling method (ESM) assessments conducted at baseline, end of therapy (16 weeks), and follow-up (28 weeks). The research questions focused on whether those in the AV-BRF or AV-EXT arms experienced less voice-related distress, anxiety, and beliefs as measured by ESM, compared to TAU. Separate mixed-effects models were fitted for each research question. The final sample (*n* = 200) completed approximately 40% of questionnaires across all timepoints. Participants who received AV-EXT therapy, but not AV-BRF, reported reduced momentary voice-related distress at 16 (*P* = .022) and 28 weeks (*p* = .029). Appraisals of voice control were also reduced in the AV-EXT arm at 16 weeks when the voice was present (*P* = .002) or not (*P* = .008). Voice power appraisals were reduced (*P* < .035) in both arms when the voice was “not present but on my mind” at all timepoints. There were no changes in the frequency of voice hearing, appraisals of voice intent, or assertive responding. These findings from everyday life, reported for the first time, provide evidence of the impact on the primary AVATAR therapy treatment targets, including appraisals of voice power and control. The weight of evidence favors the AV-EXT protocol in the further development and implementation of AVATAR therapy.

## Introduction

Voice hearing is a common experience, and although it can be positive and meaningful, many voice-hearers report persecutory and distressing voice-hearing experiences. This is common in those with a diagnosis of psychosis, with over 70% experiencing distressing voices.^1^ Distressing voices often persist for many years, despite current pharmacological and psychological interventions available, and significantly negative impact the voice-hearers’ quality of life.^2,3^ It is vital to develop effective interventions that target voice-related distress and improve outcomes for voice-hearers.

The experience of voice hearing alone is not sufficient to transition to a diagnosis of psychosis or a “need for care.”^4^ Cognitive models propose instead that beliefs, appraisals, and behavioral responses play a central role in the development and persistence of positive symptoms of psychosis,^5,6^ including voices.^7^ Important prior work demonstrated the key role of beliefs around the voices’ identity, power, control, and intent in contributing to voice-related distress.^8^ Birchwood et al. found that individuals often report their voice is more powerful *than themselves*, indicating a relational aspect to this belief.^9,10^ It has also been shown that people who hear voices have frequently experienced trauma, discrimination and/or bullying, and the distressing voice often embodies these experiences of powerlessness and subjugation in the relationship.^11^

AVATAR therapy is a relational approach, which builds on these theoretical developments using facilitated dialogs between a voice hearer and a digital embodiment (a 3D head and voice on a laptop screen) of their distressing voice (“the avatar”) in each therapy session.^12^ The therapeutic focus is on empowering the voice hearer and increasing their sense of control in the relationship with the distressing voice. AVATAR therapy shares commonalities with other relational approaches including relating therapy,^13^ voice dialogue,^14^ and talking with voices.^15^ AVATAR therapy has a growing evidence base in reducing voice-related distress following a pilot trial,^16^ and RCT with an active control^17^ as well as independent replications of the approach using immersive virtual reality technology.^18,19^ Building on this previous work, AVATAR2 is a multi-site randomized trial of AVATAR therapy, testing the delivery of 2 forms, brief (AV-BRF) and extended (AV-EXT), by a large cohort of therapists across geographically diverse sites.^20^ The primary outcome was voice distress, as measured by the Psychotic Symptom Rating Scales-Auditory Hallucinations (PSYRATS-AH),^21^ and this was significantly improved in both forms at 16 weeks, but not 28 weeks, compared with treatment as usual (TAU). Voice frequency was reduced in AV-EXT at both timepoints, and voice severity (both measured by PSYRATS-AH) improved in both versions at 16 weeks, but not 28 weeks. There were significant improvements in a range of secondary outcomes across both forms of AVATAR therapy, with AV-EXT demonstrating evidence of a more sustained impact on reduced persecutory beliefs and increased empowerment and well-being.

The emotions, thoughts, and body sensations associated with distress are context-dependent and often fluctuate from moment to moment. However, clinical trials of psychological therapies have usually relied on retrospective self-report or interview measures to assess outcomes, including distress and related experiences. Self-report and interview measures are comprehensive and standardized assessments of relevant variables but may be influenced by recall bias and social desirability in the interaction with the assessor.^22^ Experience sampling methodology (ESM) involves collecting data intensively, over time, in a real-world setting and enables us to gain insight into context-dependent experiences and responses. A recent review of 68 studies has concluded that ESM is a powerful methodological tool in studies with people with psychosis.^23^ This method has been used to understand specifically the experience of hearing voices in daily life in this population and has identified negative affect as a predictor of voice occurrence, mediated by beliefs about voice power.^24^ A further study has identified that once triggered, voice hearing maintains itself and emphasizes the importance of identifying triggers as therapeutic targets.^25^ A clear gap in the existing literature is whether distress and related beliefs are an important factor when the voice is not present, but the person is thinking about their voice, this novel area was included in our ESM questionnaire.

ESM has recently begun to be applied in clinical trials of psychological interventions in psychosis^26–28^ providing novel insights into mechanistic changes and dynamic outcomes in daily life. ESM was incorporated into the AVATAR2 trial design to complement the data reported within the primary outcome paper.^20^ The aim was to understand the potential impact of AVATAR therapy on voices assessed in the flow of daily life, with a particular focus on key targets of the therapy.^29^ Specifically, the study aimed to assess whether distress and anxiety in relation to real-time experience of voices, as well as voice presence, differ between treatment arms and over time. Secondary aims were to assess voice-related beliefs (power, control, and intent) and assertive responses to voice hearing. This is the first time ESM will be used to assess the efficacy of AVATAR therapy.

### Hypotheses

- Voice-related momentary distress will be reduced in the AV-BRF and AV-EXT arms compared to TAU alone at 16-week and 28-week timepoints.
- Voice-related momentary anxiety will be reduced in the AV-BRF and AV-EXT arms compared to TAU alone at 16-week and 28-week timepoints.
- Voices will be less likely to occur in the AV-BRF and AV-EXT arms compared to TAU alone at 16-week and 28-week timepoints.
- Appraisals of voice power, control, and intent to harm will be reduced in the AV-EXT and AV-BRF arms compared to TAU alone at 16-week and 28-week timepoints.
- Assertive responses to the voices will be higher in the AV-BRF and AV-EXT arms compared to TAU alone at 16-week and 28-week timepoints.

## Methods

### Study Design

This study includes a subsample of participants randomized into the AVATAR2 trial. AVATAR2 is a multi-site, assessor-blinded, parallel-group RCT assessing the efficacy of 2 forms of AVATAR therapy; AV-BRF (6 sessions of AVATAR therapy) + TAU, AV-EXT (12 sessions of AVATAR therapy) + TAU compared to TAU alone. The study received ethical approval (Camberwell St. Giles Research Ethics Committee: [20/LO/0657]) and was prospectively registered with the ISRCTN registry, where the trial protocol^30^ and statistical analysis plan can be accessed (ISRCTN55682735). The full results of the trial are reported elsewhere.^20^ The analysis of the ESM outcome data was pre-registered on the Open Science Framework.^31^

Patient and public involvement played a key role at all stages of the AVATAR2 trial, including design, recruitment of staff and participants, data collection, analysis, and dissemination. An active and creative group of people was established, comprising 20 members across all 4 sites, from diverse backgrounds, with lived experience of mental health conditions and recovery, including carers. The time-variant variables are included in the ESM questionnaire (Appendix), which was developed with lived experience input into the questionnaire, particularly guiding the language selected for the affect items (eg, stressed instead of anxious). The experience of completing the ESM assessments as part of the AVATAR2 trial was explored qualitatively with lived experience co-interviewers. Key themes identified included valuing regular monitoring of symptoms and related insights, as well as frustration with technical issues when they arose (S. Dennard et al., unpublished manuscript).

### Randomization and Blinding

After baseline assessment, participants were allocated in a 1:1:1 ratio to the 3 arms. Randomization, undertaken independently by the King’s Clinical Trials Unit, was stratified by site and baseline voice characterization using permuted blocks of varying sizes. Research assessors, who also supported the ESM component of the assessments, were blind to group allocation and did not have access to medical records post-randomization or the therapy database at any point.

### Participants

Potential participants were selected and contacted by a member of their clinical team at affiliated NHS sites. Other routes for participation included self-referral or contact through NHS Trusts/Institutional research registers. Eligibility criteria were then confirmed by a research assistant through consultation with the clinical team and electronic records and all participants provided informed consent. The ESM component of the AVATAR2 trial was optional, and therefore the participants in this study represent a subsample of those in the trial.

### Inclusion Criteria

Individuals were eligible to participate in the trial if they met the following criteria:

1. Over 18 years old
2. Currently experiencing frequent and distressing voices (spoken in English) persisting for ≥6 months (defined as a score of ≥1 for each item associated with the intensity of distress/voice frequency on the PSYRATS-AH^21^)
3. Currently receiving care from a specialist mental health team as an inpatient/outpatient
4. Having a diagnosis of a schizophrenia spectrum disorder (ICD-10 F20-29) or an affective disorder with psychotic symptoms (ICD-10 F30-39; subcategories with psychotic symptoms) determined using clinical records and an additional consultation with their clinical team if necessary.

### Exclusion Criteria

Potential participants were excluded from the trial if they met any of the criteria below:

1. A primary diagnosis of a substance use disorder, personality disorder, or learning disability
2. Lack of capacity to give informed consent
3. Currently attending psychological therapy targeting voices
4. Currently experiencing an acute mental health crisis
5. Profound hearing/visual impairments
6. Comprehension of English was insufficient to engage in therapy/assessment.

### Measures

Full details of the questionnaires and interview measures collected at each timepoint can be found in the trial paper^20^; this paper describes the analysis of the ESM questionnaire only. Time-invariant variables included in this study were the treatment arm (TAU, AV-BRF, and AV-EXT) and timepoint (baseline, 16 weeks follow-up, and 28 weeks follow-up). The questionnaire consisted of 34 items. Three items were forced choice (completed in every ESM assessment and resulted in branching) and 31 used a Likert scale from 1 to 7. A composite variable, voice-related momentary anxiety, was created from 4 affect items (safe/scared/relaxed/stressed) which had the following within-person reliability omega at baseline (0.015), 16 weeks (0.006), and 28 weeks (0.003), the between-person reliability omega is also reported for baseline (0.012), 16 weeks (0.006), and 28 weeks (0.014). See Table 1 for a summary of the time-variant variables included in this analysis.

**Table 1.** ESM Items and Corresponding Time-Variant Dependent Variables in Analyses

Variable/construct	ESM item	Scale/response options
Voice present	Right Now, Is your Main Voice	Present/Not Present/Not there but on my mind
Voice-related momentary distress	Right Now, My Main Voice is upsetting me	1 (Not At All)—7 (Very Much So)
Voice-related momentary anxiety	Right now, I am Safe/Scared/Stressed/Relaxed^a^	1 (Not At All)—7 (Very Much So)
Voice power	Right now, my main voice, is stronger than me	1 (Not At All)—7 (Very Much So)
Voice control	Right now, my main voice, is something I can’t control	1 (Not At All)—7 (Very Much So)
Voice intent	Right now, my main voice wants to	−7 (Hurt me)—0 (Neutral)—+ 7 (Help Me)
Assertive responding to voices	Right now, in relation to my main voice I’m standing up to it	1 (Not At All)—7 (Very Much So)

^a^The mean was taken of the 4 items to create a composite variable, with safe and relaxed reverse scored.

### Procedure

ESM assessments were completed at baseline, end of treatment (16 weeks) and follow-up (28 weeks) of the AVATAR2 trial, and consent was taken for this study as part of the AVATAR2 trial consent process. The ESM assessment period lasted 6 days and started the day after the research assessment at each timepoint of the trial. The researcher set up the M-path app^32^ on the smartphone with the participant and completed a practice questionnaire. Participants were loaned a smartphone for the study period if they were concerned about data usage or did not have access to a smartphone. Research assistants completed a check-in call on (or as close as possible to) day 2 of the ESM study period. M-path allowed the completion rate to be tracked by the researcher and if this was low (<50%), they would directly address this with the participant and resolve any issues. Participants’ individual responses were not monitored by the research team during the study period. Participants were not informed of their treatment allocation in the trial until after the conclusion of the baseline ESM study period, in case this influenced their responses and there was no overlap with the intervention period. Participants received £15 reimbursement for each ESM study period they completed during the trial.

The ESM schedule used an interval-contingent sampling scheme with semi-random intervals. The day (7.30 am-10.30 pm) was divided into 10 × 90-min blocks and each questionnaire notification (“beep”) was randomly scheduled within one of these blocks, with at least 15 minutes between each one. To ensure validity of response, the questionnaire timed out if no items had been completed within 2 minutes, or if the full questionnaire was not completed within 15 minutes.

### AVATAR Therapy

AVATAR therapy involves a person engaging in a dialogue with their dominant voice which is voiced by a trained therapist in a separate room, assisted by avatar computer software.^16^ Both AV-BRF and AV-EXT begin with an initial session where participants create an “avatar” representing their most distressing voice. In subsequent sessions, the therapist and participant begin the session by reviewing the week and previous session and then agree on a focus for the dialogue with the Avatar. The therapist then moves to a separate room and the person engages in an active 3-way dialog with the Avatar (voiced by the therapist), and the therapist. Following this dialogue, the therapist returns to the room allowing the participant to reflect on their experience. Each session lasts approximately 60 minutes with the avatar dialogue lasting approximately 10-15 minutes.

AV-BRF had a standardized focus on exposure, assertiveness, and self-esteem and AV-EXT had a phase 1 mirroring AV-BRF, augmented by a more personalized, developmentally focused phase 2, based on the voice hearer’s life history. The dialogue with the avatar in each session was recorded and shared with participants so they could listen to it again at home. Full further details on AVATAR therapy are described in the main trial paper and related resources.^20,29,33^

### Statistical Analysis

The AVATAR2 trial was powered to detect an effect size of 0.5 for the primary outcome (PSYRATS-AH Distress) in a mixed-effects (random) model at all post-randomization timepoints, in line with the multi-site design and following the large effect sizes in the AVATAR trial.^17^ ESM is a novel approach to data collection in the context of a randomized controlled trial of psychological therapy in psychosis. The recruited AVATAR2 subsample had sufficient power to test the hypotheses while minimizing convergence problems, and is a larger sample than other trials employing this methodology, for example,^27,28^ A simulation-based power analysis^34^ was performed to estimate the power for investigating differences between participants at baseline and 16 weeks in TAU compared to AVATAR-Brief if this study were to be replicated, and this was confirmed to be over 80%.

Models were fitted to see if the completion rate is predicted by demographic variables (age, gender, and ethnicity). Any demographic variables that significantly predicted compliance were included as a covariate in the main analysis.

The ESM dataset generated had a 3-level structure—repeated measurements (level 1), nested within timepoints (level 2), and nested within individuals (level 3). In R-studio, linear mixed-effects models (lme) were fitted to analyze time-variant variables. The questionnaire branched depending on whether the person indicated their main voice was present, “not there but on my mind” or not present, and separate models were therefore fitted for occasions where the voice was present or “not there but on my mind” as these included the relevant items for our hypotheses.—a: compliance (each demographic variable was a predictor in a separate model) and voice presence.

All models were fitted using restricted maximum likelihood (REML) estimation to enable analysis of all available data under the assumption that data are missing at random and all variables associated with missing values are included in the model. Each model included a treatment arm, timepoint, and a treatment × timepoint interaction as predictors, as well as a 3-level random intercept to account for the nested data structure (beep/day/person). The outcomes are reported for the interaction predictor in the model. Within each timepoint (level 2), level 1 within-person errors were modeled to have a continuous autoregressive structure (of the exponential type), with a beep number at each timepoint as a continuous time covariate. Level 1 within-person errors were allowed to be correlated between time points.

All tests were 2-tailed with an adjusted *P*-value of .035, this is to accommodate the multiple comparisons between each form of AVATAR therapy and TAU and is in line with the statistical analysis plan for the AVATAR2 trial.^20^

## Results

### Sample

Of the full sample of 345 participants recruited into the AVATAR2 trial, 209 (60.5%) consented to take part in the ESM study. These 209 participants were allocated to the 3 treatment arms as follows: TAU (*n* = 69), AV-BRF (*n* = 67), and AV-EXT (*n* = 73). The demographics of the sample can be seen in Table 2 below; there were no significant differences between the arms in the ESM study subsample. The ethnicity and gender of the sample are representative of the full AVATAR2 trial sample,^20^ but when compared with the 136 people who did not consent to the ESM study, the sample in the ESM study were significantly younger (mean in ESM = 36.94, mean in non-ESM = 43.61, *t*(287) = −4.69, *P* < .001) and started hearing voices at a younger age (mean in ESM = 23.23, mean in non-ESM = 26.85, *t*(278) = −2.8, *P* < .01).

**Table 2.** Demographics of the ESM Sample (*n* = 209) by Arm

Demographic variable	AV-EXT (*n* = 73)	AV-BRF (*n* = 67)	TAU (*n* = 69)
Age mean (SD)	37.82 (13.74)	36.49 (12.54)	36.65 (12.24)
Age AH started mean (SD)	25.65 (12.04)	21.42 (10.3)	22.43 (11.18)
Gender (%)			
Female	39.7	41.8	42
Male	58.9	58.2	55.1
Other	1.4	0	2.9
Living situation (%)			
Alone	42.5	49.3	43.5
Partner	5.5	4.5	11.6
Spouse	2.7	7.5	7.2
Parents	32.9	25.4	21.7
Other relatives	4.1	0	2.9
Others	12.3	11.9	13
Psychiatric diagnosis (%)			
F20 Paranoid schizophrenia	34.2	41.8	43.5
F22 Persistent delusional disorder	0	1.5	1.4
F23 Acute and transient psychotic disorder	2.7	0	1.4
F24 Induced delusional disorder	0	1.5	0
F25 Schizoaffective disorder	11	7.5	7.2
F28 Other nonorganic, psychotic disorder	0	1.5	0
F29 Unspecific nonorganic psychosis	43.8	32.8	30.4
F31 Bipolar affective disorder	1.4	1.5	4.3
F32.3 Severe depressive episode with psychotic symptoms	6.8	11.9	8.7
Not available	0	0	2.9
Level of education (%)			
Primary School	2.7	1.5	0
Secondary School (no exams)	15.1	11.9	15.9
Secondary School (O/CSE equivalent)	1.4	6	8.7
Secondary School (A Level equivalent)	24.7	14.9	18.8
Vocational Education/College	31.5	32.8	39.1
University Degree/Professional Qual	23.3	31.3	17.4
Not available	1.4	1.5	0
Ethnicity			
Black African	9.6	13.4	5.8
Black Caribbean	6.8	3	7.2
Black-Other	1.4	4.5	2.9
Chinese	0	1.5	0
Indian	1.4	3	4.3
Other	17.8	14.9	14.5
Pakistani	2.7	3	1.4
White	60.3	56.7	63.8
Employment status			
Full-time	8.2	10.4	11.6
Part-time	6.8	3	10.1
Housewife/husband	1.4	1.5	2.9
Self-employed	2.7	0	0
Student	9.6	11.9	11.6
Unemployed	69.9	73.1	63.8
Not available	1.4	0	0

### Data Availability

Of the 209 people who consented to take part in the ESM study, 200 went on to provide ESM data. Those who provided no responses were excluded from the analyses, but no further thresholds for inclusion were set. Of these 200 participants, 83 (41.5%) provided data at all 3 timepoints, 67 (33.5%) at baseline only, 28 (14%) at baseline and 16 weeks, 16 (8%) at baseline and 28 weeks, and a small number completed only 16 weeks (*n* = 2), 28 weeks (*n* = 2), or 16 + 28 weeks (*n* = 2). Completion rate was defined as the number of questionnaires completed at each timepoint as a percentage of the total available (*n* = 60). The mean completion rate at each timepoint was (40.9%, SD = 27.8) at baseline, 41.1% (SD = 28.0) at 16 weeks, and 38.1% (SD = 28.1) at 28 weeks. Timepoint significantly predicted the completion rate (*P* = .0019). None of the demographic variables specified (age, gender, or ethnicity) predicted the completion rate, and neither did voice severity as measured by the total score on the PSYRATS-AH. The total data available at each timepoint (each study period was 6 days) is outlined in Table 3.

**Table 3.** Data Availability (Number of Questionnaires/Beeps Completed) by Timepoint and Treatment Arm

	Baseline (*N* = 194)	16 weeks (*N* = 105)	28 weeks (*N* = 103)
TAU	1699	936	699
AV-BRF	1475	899	818
AV-EXT	1565	994	840

### Analysis of Outcomes

#### Momentary Voice-Related Distress and Anxiety

Momentary voice-related distress when the voice is present (see Table 4 for data availability across categories of voice presence) was significantly reduced in the AV-EXT arm, compared to TAU at 16 weeks (β = −0.72, SE = 0.33, *t*(148) = −2.2, *P* = .029) and 28 weeks (β = −0.86, SE = 0.37, *t*(148) = −2.31, *P* = .022). There were no significant reductions in AV-BRF compared to TAU at either timepoint.

**Table 4.** Availability of Data Across the 3 Categories of Voice Presence

	Voice present	Not there but on my mind	Voice not present
Baseline	2281	989	1469
16 weeks	1194	427	1209
28 weeks	979	301	1079

Momentary voice-related distress, when the voice is “not there but on my mind,” was not significantly reduced in AV-EXT or AV-BRF compared to TAU at either timepoint, the effect fell just outside the a priori threshold of *P* = .035, for AV-EXT at 28 weeks (β = −0.87, SE = 0.42, *t*(111) = −2.05, *P* = .043).

There were no significant reductions in anxiety, when the voice was present, across treatment arms or timepoints. Anxiety, when the voice is “not there, but on my mind,” was significantly reduced in AV-EXT compared to TAU at 16 weeks (β = −0.59, SE = 0.24, *t*(111) = −2.42, *P* = .02). There were no other significant changes in the therapy arms over time for when the voice is “not there, but on my mind.”

No distress or anxiety outcomes were moderated by demographic or clinical variables (gender, ethnicity, and duration of voice hearing).

#### Voice Presence

The distribution of voice presence across each timepoint can be seen in Table 4:

Treatment arm did not significantly predict voice presence moment-by-moment, and there were no interactions with timepoint.

#### Voice Appraisals

##### Voice Power

Appraisals of voice power, when the voice was present, were not significantly reduced in the AV-EXT or AV-BRF arm compared to TAU across timepoints.

Appraisals of voice power were significantly reduced in AV-EXT compared to TAU when the voice was ‘not there but on my mind’ at 16 (β = −1.05, SE = 0.37, *t*(111) = −2.85, *P* < .01) and 28 (β = −0.88, SE = 0.41, *t*(111) = −2.15, *P* = .03) weeks. Appraisals of voice power were also significantly reduced over time compared to TAU in AV-BRF at 16(β = −0.94, SE = 0.41, *t*(111) = −2.32, *P* = .022) and 28 (β = −1.05, SE = 0.46, *t*(111) = −2.27, *P* = .025) weeks. These findings were not moderated by gender, ethnicity, or duration of voice hearing.

##### Voice Control

Appraisals of feeling controlled by the voice, when the voice is present, were significantly reduced in the AV-EXT arm compared to TAU at 16 weeks only (β = −0.99, SE = 0.32, *t*(148) = −3.1, *P* < .01). There were no significant reductions in AV-BRF compared to TAU. This finding was significantly moderated by gender, with a significant 3-way interaction term (β = −1.5, SE = 0.64, *t*(139) = −2.36, *P* = .02) and a stronger relationship in females, but not ethnicity or duration of voice hearing.

Appraisals of feeling controlled by the voice, when the voice was “not there but on my mind,” were also significantly reduced in AV-EXT at 16 weeks only (β = −0.97, SE = 0.36, *t*(111) = −2.7, *P* < .01); there were no significant improvements in AV-BRF compared to TAU. This finding was not moderated by gender, ethnicity, or voices duration.

##### Voice Intent

There were no significant effects for any timepoint and treatment interaction terms in the models predicting voice intent, either when the voice is present, or “not there but on my mind.”

#### Assertive Responding to Voices

There was no increase in assertive responses to voices, when the voice is present, or “not there but on my mind,” in either treatment arm compared to TAU, over time.

## Discussion

This study represents the first application of ESM to test the impact of AVATAR therapy (in brief and extended forms) in the flow of daily life. The distress a person hears in response to a voice is a key target of AVATAR therapy, and the AVATAR2 trial found significant reductions in distress (PSYRATS-AH) at 16 weeks in both AV-BRF and AV-EXT, compared to TAU, but not at 28 weeks. The findings of this ESM study suggest that people who received the extended form of the therapy experienced less distress related to their voice in daily life at 16 weeks, and unlike the PSYRATS-AH findings, this was maintained at 28 weeks. A further difference with the PSYRATS-AH findings was that the ESM outcomes did not show significant change in AV-BRF at either timepoint. However, it would be premature to draw conclusions about differences in sensitivity to change across the 2 measures. The PSYRATS-AH distress subscale is a multidimensional measure of distress (amount, intensity, content, and controllability) and asks for the person’s experience across the previous week. The ESM item is a single question and asks about the person’s experience “right now.” It is perhaps not that one is more sensitive to change in distress, but different aspects of voice-related distress are represented in these complementary findings. Furthermore, it seems that whilst participants in the AV-BRF arm did report a significant reduction in distress at 16 weeks when asked to rate their experiences over the previous week in the PSYRATS-AH interview, this was not replicated in the distress experienced when the voice was present as measured in the ESM assessments.

Voice frequency was also assessed with the PSYRATS-AH in the AVATAR2 trial and showed sustained improvements in the AV-EXT arm. This is a very different measure from the model constructed in the ESM analysis which predicted occurrence moment-by-moment, rather than an overall measure of voice frequency, which explains the contrasting findings across these approaches, with no change in voice occurrence in this ESM study. There were changes at both timepoints across stress, anxiety, and depression self-report measures in the AVATAR2 trial, particularly for AV-BRF but voice-related anxiety did not show changes in daily life, with the exception of reduced anxiety in AV-EXT at 16 weeks only when thinking about the voice (“not there but on my mind”).

Appraisals relating to voice power and control are potential mechanisms that contribute to voice-related distress^8^ and therefore are key targets of AVATAR therapy. These findings showed the person felt more in control of their main distressing voice in their daily life, at the end of therapy in the AV-EXT arm both when the voice is present, and when it is “not there but on my mind.” The people in both therapy arms report that their voice has less power at the end of therapy, and at follow-up compared to TAU, but only when it is on their mind and not when it is present. Convergent findings from self-report measures of voice power in the AVATAR2 trial (Beliefs About Voices Questionnaire-Revised Omnipotence [BAVQ-R] and Voice Power Differential Scale [VPDS)]^9,35^ showed change in AV-EXT only at both 16 and 28 weeks. These findings suggest AVATAR therapy is effectively targeting these appraisals, and the conviction in the beliefs about voices is reducing but also fluctuates depending on whether the voice is present. Specifically, it seems that appraisals of voice power and control do reduce during therapy, but this change is seen more readily when the voice is not present but still being thought about by the person. It appears conviction in appraisals of voice power and control remain stronger when the voice is present, despite these reductions when the person is thinking about their voice.

It is noteworthy that voice-related distress is reduced at 28 weeks in the absence of sustained reductions in appraisals of voice power and control when the voice is present (although these are reduced when the voice is on their mind). Other aspects of distressing voice hearing were assessed in the ESM questionnaire (including voice content, loudness, and disruption), and we plan to examine research questions related to these outcomes, and potential mechanisms, in subsequent analyses as outlined in our pre-registration. Consultation with Experts by Experience (as outlined in the AVATAR2 paper^20^) highlighted priority outcomes for them, including a life less affected by voice hearing and to be at peace with voice hearing. It may be that a cognitive shift in beliefs about power and control when the voice is on their mind contributes to these outcomes, but this cognitive shift is more difficult to maintain when the voice is present. However, the fact it has occurred and may revert quickly when the voice is not present has implications for the person’s wider ability to cope. It may also suggest, given the wider improvements in distress and other secondary outcomes reported in this paper, and the AVATAR2 paper, that beliefs about power and control of the voice do not need to be the central mechanisms of change in psychological therapy for distressing voices. This work highlights the importance of measuring and testing several mechanisms of change in psychological therapy trials and ESM can be a useful method to do so, particularly where the mechanisms pertain to processes that are hypothesized to occur in the flow of daily life, or “in the moment” rather than broader appraisals or experiences. Further work planned by the AVATAR team includes mediation analyses of the AVATAR2 outcomes, incorporating self-report measures of voice power (VPDS), control, and intent (BAVQ-R) which will further inform this question.

The intent of the voice did not show change, and it may be these beliefs are difficult to disentangle from voice power; indeed, a factor analysis of the BAVQ-R found that items measuring malevolence (intent), and omnipotence (power) loaded onto a single factor.^36^ It seems that people who receive AVATAR therapy may not change their beliefs around voice malevolence/benevolence, although may come to perceive the voice to have less power and influence over them. This is occurring in the context of reduced beliefs about wider persecution in the AV-EXT group when assessed by the PSYRATS-DEL^21^ interview measure in the AVATAR2 trial. Reductions in voice control were moderated by gender, suggesting female participants experienced a larger reduction in voice control than male participants; this was not found in the wider AVATAR2 trial data and is an intriguing finding that warrants replication.

The ESM questionnaire assessed several ways of responding to the voices, both when they are present and when thinking about them. This initial ESM study reports the findings on assertive responding, and there were no improvements in this seen in either therapy arm. On the one hand, this could suggest potential challenges in generalization to the daily life of an aspect of therapy that is prominently associated with the AVATAR therapy approach. However, an evolution in the understanding of AVATAR therapy since it was first developed, has seen the therapeutic focus shift from assertiveness to empowerment. While assertive responding is often foregrounded in early therapy dialogs, what an empowered stance might involve in the flow of daily life is likely to be more context-dependent. Moment-to-moment assertive responding brings an attendant risk of maintaining attentional focus on the voice rather than current activity and therefore discussions around generalization to situations outside of sessions typically focus on the person taking control over their actions. As such, measures such as the Voice Acceptance and Action Scale (VAAS),^37^ which assesses the person’s ability to respond mindfully to the voice and act in line with personal values, may more closely tap into empowered relating that is a key target of the therapy. Notably sustained effects on VAAS were found for both AV-EXT and AV-BRF in the main trial paper.^20^

As we have outlined above, the ESM findings do show some divergence from the AVATAR2 trial findings, particularly regards the impact of AV-BRF therapy. This highlights that ESM offers novel insights alongside self-report and interview measures and therefore is a valuable addition to outcomes in clinical trials. The interpretation is complex, particularly where constructs overlap, for example, voice-related distress on PSYRATS-AH and ESM, and further studies adopting both ESM and retrospective measures as outcomes are needed to understand this. We hope to contribute by pooling data and examining relationships between ESM and other outcome measures in this trial and others as more adopt this approach. While there are some indications of the positive impact of AV-BRF, consistent with the main trial findings, the results in daily life favor AV-EXT. This adds to the balance of the results in the AVATAR2 trial and supports the recommendation that the further development and implementation of AVATAR therapy should be primarily guided by the AV-EXT protocol.

### Limitations

This ESM study uses a subsample of participants from the AVATAR2 trial and therefore does not reflect the entire trial sample and is open to potential selection bias, although participants opted into the ESM study before knowledge of their randomized allocation. The ESM subsample were younger, and started hearing voices at a younger age, than those who did not consent to the ESM substudy in the AVATAR2 sample. This may limit the generalizability of the findings to older people with psychosis and to those with voice hearing onset in mid-life or beyond. The completion rates across the ESM assessment period are below 50%, and while this is similar to other studies in this population^23^ and considered sufficient for the analyses performed, it is lower than another recently published paper reporting ESM outcomes of a psychological therapy trial^27^ and should be noted when interpreting the findings. Tolerability of ESM as an outcome measure is an important question to consider as this approach is rolled out more widely. Participants in the AVATAR2 trial did not receive substantial additional reimbursement (£15 at each timepoint), and the ESM was introduced at the end of a lengthy clinical assessment meaning limited time and energy for a detailed briefing. In future, we would consider offering greater incentives and completing the briefing at another time or earlier in the clinical assessment to improve completion rates. There were no demographic variables identified that contributed to missingness but there is a possibility of nonrandom missingness that we have not accounted for. The ESM questionnaire was grounded in existing interview and questionnaire measures (eg, PSYRATS-AH and BAVQ-R) but has not been validated so items may not reliably assess the constructs they were intended to measure. The items have been entered into the ESM item repository (https://osf.io/kg376/) to support efforts to validate questionnaires in the future.

## Conclusion

ESM data enable the examination of psychological therapy outcomes in everyday life, where every therapist hopes to make an impact. ESM is a valuable addition to the assessment of outcomes in clinical trials and has allowed us to examine key therapeutic targets in daily life and capture novel insights regarding where change might primarily occur in the windows of time when voices are not present, but the person is thinking about them. Qualitative work completed as part of this study highlighted the significant benefits reported by participants, including finding it useful to reflect on their own experiences through completing the ESM study.^38^ The findings of this study suggest that AVATAR therapy, particularly AV-EXT, can have a positive impact on everyday life, but further research is required to understand the specific changes occurring, and the wider implications. These findings add to the evidence generated in the multi-center AVATAR2 trial and further support the recommendation that the AV-EXT protocol should guide the provision of AVATAR therapy as it is developed and more widely implemented.

## Figures and Tables

**Figure 1.**
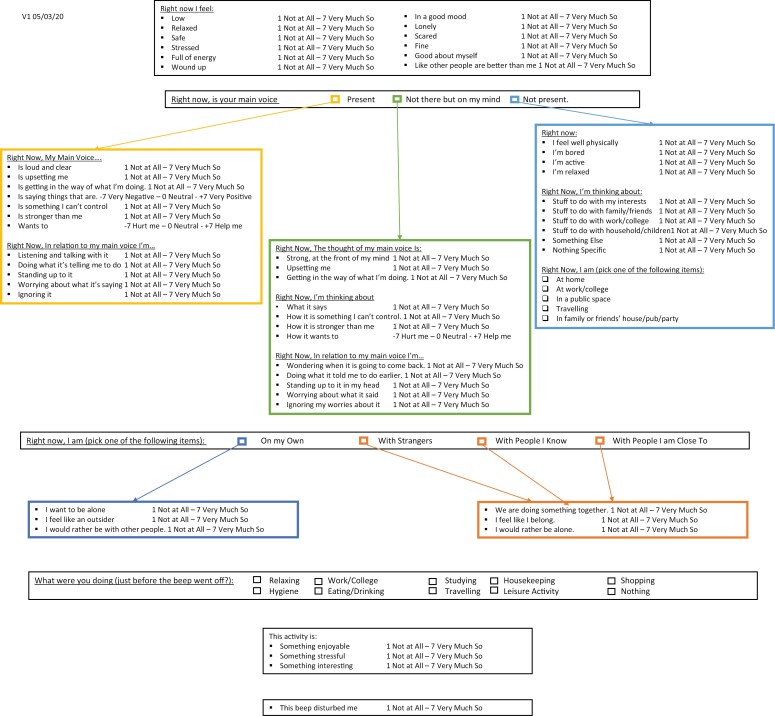
Full ESM Questionnaire
